# Single-stranded DNA aptamer-based rolling circle amplification as anti-chicken *Salmonella* bacteriostatic

**DOI:** 10.14202/vetworld.2022.1171-1176

**Published:** 2022-05-11

**Authors:** Samer Sadeq Hameed, Adil Sabr Al-Ogaili, Noor Noori

**Affiliations:** 1 Department of Pathology and Diseases of Poultry, College of Veterinary Medicine, University of Baghdad, Baghdad, Iraq; 2 Department of Medical Laboratory Techniques, Kut-Technical Institute, Middle Technical University, Baghdad, Iraq

**Keywords:** DNA aptamers, rolling circle amplification, *Salmonella*

## Abstract

**Background and Aim:**

*Salmonella* is a major foodborne pathogen in the poultry industry, wherein the control measures may include sanitation and antibacterial and vaccines. However, there have been severe global restrictions on using anti-*Salmonella* antibacterial agents in livestock. This situation, along with rapidly increasing drug-resistant bacterial species, has led to the exploration of unconventional methods to control *Salmonella* infection in poultry. In recent years, selection techniques of promising DNA aptamers have begun to permeate several medical branches, resulting in the development of numerous anti-*Salmonella* DNA aptamers, most of which are used as sensing molecules for diagnostic purposes. These DNA aptamers have been demonstrated to interfere with bacterial growth, multiplication, and viability. Aptamers formed in rolling circle amplification products (RCA-p) could improve the potential action of aptamer interference with bacteria. This study aimed to test the use of single-stranded DNA aptamers in the form of RCA-p as a bacteriostatic to *Salmonella* *in vitro*.

**Materials and Methods:**

*Salmonella* Typhimurium and *Salmonella* Enteritidis isolates were subjected to the action of anti-ST and anti-SE DNA aptamers in the form of RCA-p. Each isolate was grown on MacConkey and Luria-Bertani agar media separately in different concentrations in the presence or absence of the cognate RCA-p.

**Results:**

The anti-*Salmonella* species DNA aptamer-based RCA-p were capable of reducing bacterial growth to significant levels *in vitro*.

**Conclusion:**

We describe a potential solution for the rapidly developing drug resistance of several bacterial species. Our findings suggested that the use of non-toxic, non-immunogenic, and low-cost DNA aptamers targeting *Salmonella* in the form of RCA-p could inhibit the bacterial growth rate. Unlike polymerase chain reaction, RCA yields tandem repeats of single-stranded DNA at isothermal conditions, which would increase the probability of receptor-ligand clustering and increase affinity. Furthermore, as our RCA template was bivalent with two DNA aptamer sequences, we could target multiple sites or antigens on a bacterial cell.

## Introduction

*Salmonella* remains the major bacterial species among foodborne pathogens. *Salmonella* Typhimurium (ST) and *Salmonella* Enteritidis (SE) are the primary species among foodborne contaminants worldwide [1,2]. These two species, which are the non-typhoid species of *Salmonella*, are responsible for causing 95% of salmonellosis cases in humans due to the consumption of contaminated chicken meat and eggs [3,4]. Infection caused by non-typhoid *Salmonella* species is considered a zoonotic disease thereof. However, animals, including poultry, rarely develop clinical illness when infected with these bacterial species. In recent years, due to the restriction placed on the use of antibacterial drugs in birds raised for human consumption and the increasing number of highly drug-resistant bacterial species, unconventional methods are being explored for controlling ST and SE infections [5].

Aptamers are a new emerging group of molecules, which are either peptide aptamers or nucleic acid aptamers. DNA aptamers are single-stranded and promising molecules in several fields of medicine. DNA-based aptamers have been successfully demonstrated to exhibit high affinity and specificity for several bacterial antigens [5,6]. The traditional method for developing aptamers is known as SELEX, which is defined as the systemic evolution of ligands by exponential enrichment [7]. However, this method has the limitation of being labor-intensive and time-consuming when used to develop aptamers to the target molecule. Recent developments in aptamer selection methodologies have shed new insights into the beneficial use of aptamers. Today, aptamers are beginning to replace monoclonal antibodies in several branches of biology and medicine [8-10].

In recent years, several researchers have described highly efficient DNA aptamers selected toward ST and SE [5,11,12]. Although most DNA aptamers have been developed for diagnostic purposes, these aptamers might be effective in interfering with the multiplication of these bacterial species *in vitro* and *in vivo* [13]. We hypothesized that the utilization of these aptamers in the form of rolling circle amplification products (RCA-p) could increase the bacteriostatic effect of single aptamers. Our hypothesis was based on the fact that RCA yields tandem repeats of the original DNA aptamer [14].

This study aimed to use complementary sequences of recently described (anti-ST and anti-SE) DNA aptamers as a template to develop RCA-p. We also confirm that RCA-p is capable of reducing the growth of both ST and SE *in vitro* at certain concentrations.

## Materials and Methods

### Ethical approval

The study and all tests and procedures were approved by the scientific and animal care committee in the Department of Pathology and Poultry Disease in the College of Veterinary Medicine, University of Baghdad (Approval No. 26 VET/2021).

### Study period and location

The study was conducted from May 2020 to February 2021. The RCA preparation and testing were conducted in the Department of Medical Laboratory Techniques, Kut Technical Institute, Middle Technical University. All the bacterial work was done in the Department of Pathology and Diseases of Poultry, College of Veterinary Medicine, University of Baghdad.

### Bacterial isolates and culturing

The two bacterial isolates of ST and SE were a kind gift from Dr. Young Min Kwon, University of Arkansas, USA. The bacterial isolates were cultured in Luria-Bertani (LB) broth (Sigma-Aldrich, USA) at 37°C for 24 h with half aeration and shaking. Bacterial colony-forming unit (CFU) was calculated by transferring the bacterium inoculum into a series of test tubes containing sterile 1× phosphate-buffered saline (pH 7.2) to prepare tenfold serial dilutions. The 10-fold serially diluted bacterial growth was mixed with RCA-p in a final concentration of 100 μg.mL^−1^. The RCA-p-bacterial mixture was incubated at 25ºC for 2 h with shaking, after which 100 μL of this mixture was transferred onto MacConkey and LB agar media (HiMedia Lab. Pvt., India) in triplicates separately, followed by incubation at 37°C for 24 h. As a control group, plates in triplicates with bacterial inoculums and without RCA-p were incubated under the same conditions for every concentration.

### Single-stranded DNA aptamer sequences, RCA primers, RCA design

The anti-*Salmonella* DNA aptamer designs have been described previously [13]. Two DNA aptamer sequences were selected for this study. The complementary sequences of the two aptamers were incorporated in one linear sequence and separated by a 15-mer spacer. The spacer region allowed applying rigid, double-stranded DNA and non-functional region over the stretches of RCA-p. The RCA template was ordered as phosphorylated at the upstream> (IDT, Coralville, Iowa, USA) to facilitate ligation. The complementary templates of the anti-*Salmonella* aptamers were flanked by upstream and downstream primer-binding sites (Table-1) [13]. The primer measuring 20 nucleotides in length was designated to navigate the two extremities of the RCA template. The aim of the primer design and the ligation process was to circularize the RCA template. The RCA-p were annealed with a 15-mer complementary spacer. All oligos were obtained from Integrated DNA Technologies Inc. (IDT).

**Table 1:** Primer, RCA template, and spacer complementary sequences designs. The RCA template which is composed of the complementary sequences of the anti-ST and anti-SE aptamers[13] is flanked primer-binding sites and spacer region. Of course, the RCA template was ordered as phosphorylated to facilitate the T4 DNA ligase (NEB, Cat# M0202S). The primer was designed to span the primer-binding sites of the two extremities of the RCA template.

Oligo	Sequence (5’→3’)
Primer	GTTCAGATGCAAGACGTTCC
Spacer	GATCCACCGGTAGCA
RCA template	5Phos/GCATCTGAAC [AATCGCTGAATGAAATCCACGTGCGGGACAGAGGCAGTGCGTGGCGTGCGGTGAGAGGTGGATTGTGCCTC GCGAATCTGTCCTGGACTG] TGCTACCGGTGGATC [AATCGCTGAATGAAATCCACGTGGATAGCGATGTTGATAGAGCGTGAGTTGGTCGTGTTTGGAGTGGGCT CGCGAATCTGTCCTGGACTG] GGAACGTCTT

RCA=Rolling circle amplification

### RCA technique and mixing with bacterial isolates

The isothermal RCA technique and protocol were used as described by Al-Ogaili *et al*. [10]. Briefly, 5' phosphorylated RCA template (equivalent to 2 pmol) was mixed with the primer (equivalent to 1 pmol) in 1×TE buffer (Promega, Cat# V6231, WI, USA). The template-primer mixture was annealed by thermal treatment (at 95°C for 5 min and 56°C for 1 min). This was followed by the ligation process with T4 DNA ligase (NEB, Cat# M0202S MA, USA). The ligation incubation condition was overnight at 4°C as recommended by the manufacturer. Isothermal ɸ29 DNA polymerase with master mix (NEB, Cat# M0269L MA, USA) was used as the polymerization enzyme. The polymerization incubation condition was 30°C for 16 h [10]. After polymerization, the complementary spacer was added and annealed thermally (at 95°C for 5 min and 56°C for 1 min). After each enzymatic treatment, we purified and precipitated the DNA by the ethanol precipitation method. The RCA-p were mixed with the bacterial isolates separately at a final concentration of 100 μg.mL^−1^. As mentioned earlier, the bacterial isolate was 10-fold serially diluted using 0.9% N.S. The RCA-p-bacterial isolate mixture was incubated at RT for 2 h with shaking and then transferred onto LB or MacConkey agar media (HiMedia), followed by overnight incubation at 37°C.

### Statistical analyses

Statistical analyses of bacterial growth were performed using multiway analysis of variance (ANOVA) to demonstrate variation between treatments using the general linear model procedure of the SAS software [JMP Pro 13, free online software, (https://www.jmp.com/en_nl/software/predictive-analytics-software.html)]. In all data sets, mean±standard deviation and p-value were the primary comparison elements. The Tukey honestly significant difference test was used to determine the significant differences among mean values at p<0.01.

The relative level of the CFU of the RCA-p-treated inoculums was normalized by calculating the number of colonies on plates to the mean value of the non-RCA-p-treated bacterial growth as the negative control (S–N). The average mean of the readings of the non-treated bacterial inoculum was the baseline to calculate the S–N values of all traits. ANOVA with the least significant difference of the mean of the S–N values and Student’s t-test were performed to determine the differences between the treatments. Data were analyzed using the JMP^®^ software (SAS Institute Inc., Cary, NC, USA).

## Results

### RCA-p

**Figure-1:**
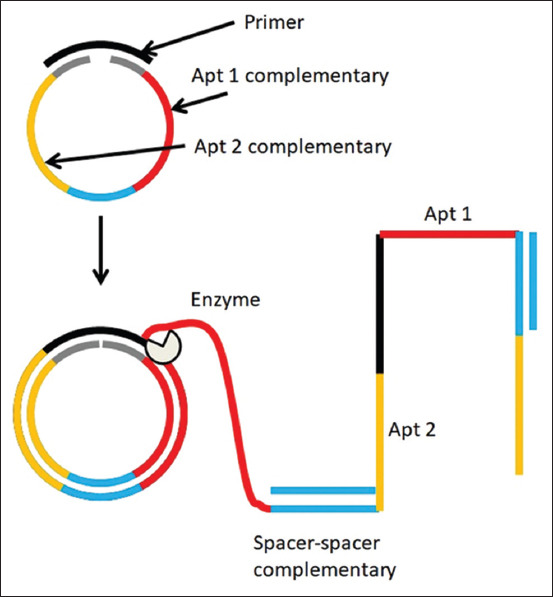
Rolling circle amplification technique. Template is designed to have the complementary sequences of aptamers 1 and 2 (Apt1 and Apt2) with primer-binding site and spacer. When the enzyme rolls on the template during the amplification process, it creates tandem repeats of the aptamers. However, spacer complementary sequence is added to create rigid, double-stranded DNA stretches along the rolling circle amplification products.

RCA-p are the tandem repeats of the template. The template was the complementary sequence of two DNA aptamers. However, the two DNA aptamers were separated by a spacer sequence. The spacer sequences along the RCA provide double-stranded DNA stretches that represent rigid areas where no activity is required (Figure-1).

### Bacterial growth

The two bacterial isolates were tenfold serially diluted in broth. The bacterial isolates exhibited small rounded opaque colonies on a solid medium at higher dilutions (1×10^−4^, 1×10^−5^, and 1×10^−5^ CFU).

### Bacterial growth on LB agar

**Figure-2:**
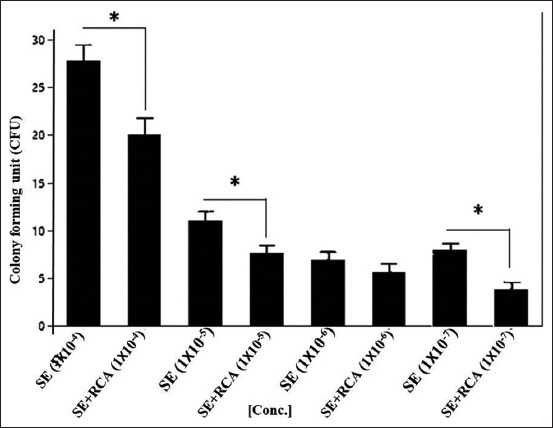
*Salmonella* Typhimurium (ST) on Luria-Bertani agar. ST isolate was 10-fold serially diluted and mixed with a constant concentration of anti-*Salmonella* DNA aptamers in the form of rolling circle amplification. The colony-forming unit was calculated and blotted with a concentration of the rolling circle amplification (RCA) products. The RCA products showed a significant ability to reduce the growth of the bacteria (p<0.01) except in 1×10^–6^ where the reduction was not significant.

**Figure-3:**
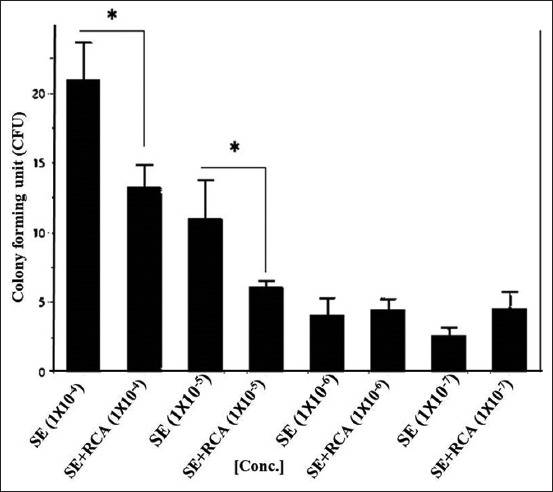
*Salmonella* Enteritidis (SE) on Luria-Bertani (LB) agar. The bacterial isolate in broth was 10-fold serially diluted and mixed with a constant concentration of anti-*Salmonella* DNA aptamers in the form of rolling circle amplification. The colony-forming unit on the LB agar was calculated and blotted with a concentration of the rolling circle amplification (RCA) products. The RCA products showed a significant ability to reduce the growth of the bacteria (p<0.001) in the higher bacterial concentrations, that is, 1×10^–4^ and 1×10^–5^.

When the ST isolate was mixed with RCA-p, there was a significant reduction in bacterial growth (p>0.01) in all samples compared with growth in the control group, except at the concentration of 1×10^−6^. Remarkably, the growth of the SE isolate was significantly reduced by RCA-p at high bacterial concentrations (Figures-2 and 3). However, at lower bacterial concentrations, the addition of RCA-p did not significantly reduce the bacterial growth.

### Bacterial growth on MacConkey agar

**Figure-4:**
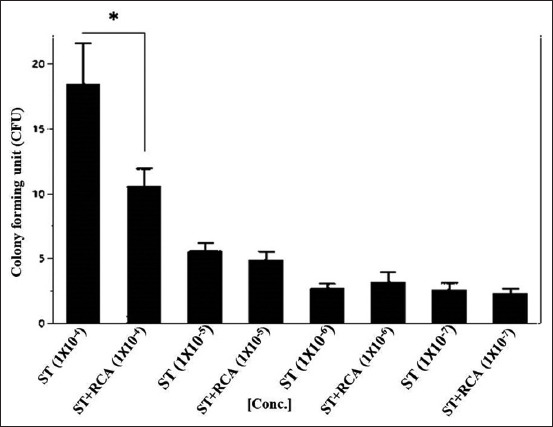
*Salmonella* Typhimurium (ST) on MacConkey agar. ST isolate in broth was 10-fold serially diluted and mixed with a constant concentration of anti-*Salmonella* DNA aptamers in the form of rolling circle amplification (RCA) products. The colony-forming unit (CFU) was calculated and blotted with a concentration of the RCA products. The CFU was significantly reduced (p<0.001) in the lower dilution 1×10^–4^.

**Figure-5:**
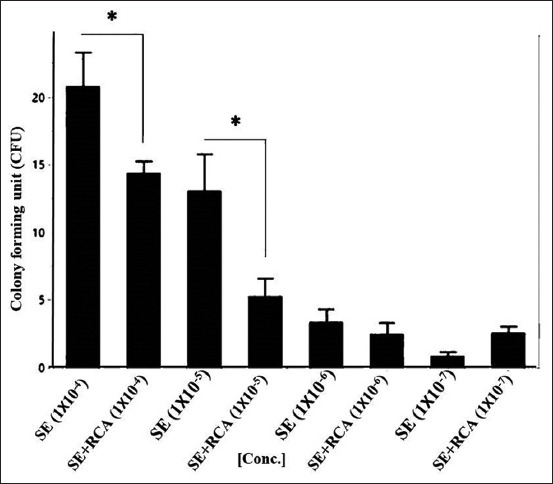
*Salmonella* Enteritidis (SE) on Luria-Bertani (LB) agar. The bacterial isolate in broth was 10-fold serially-diluted and mixed with a constant concentration of anti-*Salmonella* DNA aptamers in the form of rolling circle amplification. The colony-forming unit on the LB agar was calculated and blotted with a concentration of the rolling circle amplification (RCA) products. The RCA products showed a significant ability to reduce the growth of the bacteria (p<0.001) in the higher bacterial concentrations, that is, 1×10^–4^ and 1×10^–5^.

Regarding the growth of the bacterial isolates on MacConkey agar, the addition of RCA-p significantly reduced the growth at only higher bacterial concentrations (Figures-4 and 5). This was consistent with the fact that the relatively high concentration of RCA-p compared to the bacterial concentration in the medium did not alter the bacterial growth. As in the disk diffusion test, we did not prepare a standard McFarland bacterial suspension; therefore, the precision of the bacterial growth inhibition was not highly accurate [15,16].

## Discussion

Foodborne pathogens are a growing hazard in the chain of poultry production [17]. ST and SE are the major bacterial species on the list of foodborne contaminants worldwide [1,2]. In 2010, gastroenteritis due to non-typhoid *Salmonella* accounted for an estimated 93.8 million cases per annum [18]. In the United States, 95% of foodborne pathogens consist of non-typhoid *Salmonella*. In recent years, there has been an increasing demand for “antibiotic-free” or “organic” poultry and poultry products [19]. Aptamers (DNA or RNA) are emerging molecules that are primarily used for detection and biosensing. Although DNA aptamers offer a reliable and trusted method for controlling bacterial growth, they exert a limited effect on bacterial multiplication due to their small molecular weight.

### RCA-p

Due to the small size of the DNA aptamer compared with the bacterial cell size, aptamer interference with the bacterial multiplication process is uncertain. To overcome this limitation and increase the probability of DNA aptamer-bacterial growth interference, we suggest the development of DNA aptamers in the form of RCA-p that represent tandem repeats of the original single aptamers (Figure-1) [14]. This will increase the probability of having multiple aptamers directed to the same target. However, raw RCA-p may self-hybridize because they are single-stranded DNA [20]. This will reduce the quality of RCA-p, prevent the action of RCA-p, and decrease the probability of RCA-p-bacterial multiplication interference. Therefore, we aimed to insert a spacer in our RCA template design. The spacer provides rigid inert areas (double-stranded DNA) along the tandemly repeated flabby aptamers (single-stranded DNA) [21].

**Figure-6:**
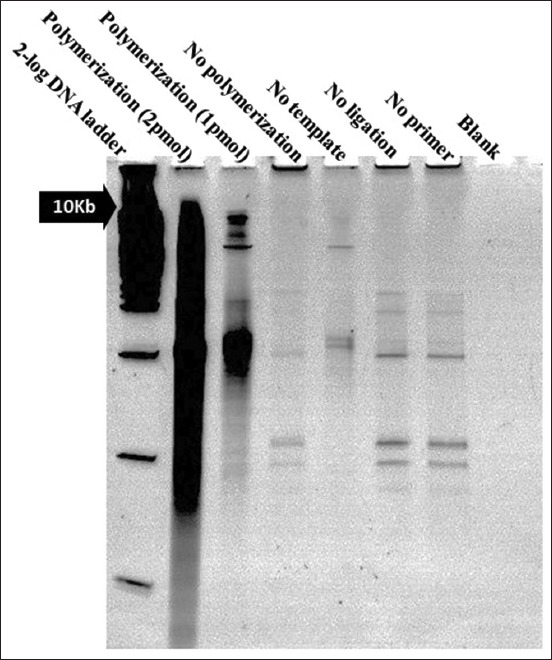
Rolling circle amplification products on denaturing 15%TBE urea gel. The templates were annealed with a single spanning primer. The ligation process with T4 DNA ligase (NEB, Cat# M0202S) was mediated by the phosphorylated upstream template. Last, ɸ29 DNA polymerase was used to polymerize the oligos (NEB, Cat# M0269L). The rolling circle amplification (RCA) products were hybridized with spacer complementary to produce tandem repeats of ssDNA alternating with dsDNA to increase the chance of interaction with target Salmonella. Few controls have been added to ensure the quality of the RCA products.

The process of RCA production involves several enzymatic treatments in exchange with DNA purification and reconcentration. Therefore, several controls must be included to exclude errors (Figure-6). Our results demonstrated that the DNA product (RCA) had a very high molecular weight (>10 kb on 15% TBE denaturing urea gel [Thermo Fisher Scientific, United States]) (Figure-6). This result was highly consistent with other RCA results [22,23]. In general, RCA-p appears as a lane smear on the denaturing gel [24,25], and this fact has been confirmed in our results also. The smearing of RCA-p on the denaturing gel is due to the single-stranded nature of RCA-p and the different lengths of the products.

### Bacterial growth

We could not include the results obtained using very high bacterial concentrations (i.e., net growth samples, 1×10^−1^, 1×10^−2^, and 1×10^−3^ CFU) because of non-countable, very dense bacterial growth at these concentrations. These densely growing bacteria were identical for both bacterial isolates and on both agar media.

#### Bacterial growth on LB agar

After mixing with RCA-p, 100 μL of both ST and SE isolates was inoculated on LB agar in tenfold serial dilutions. RCA-p were added in a constant concentration (~100 μg.mL^−1^ final concentration). For the ST isolate, the results demonstrated a significant reduction in bacterial growth (p>0.01) in all samples, except at the concentration of 1×10^−6^. Similarly, for the SE isolate, the growth was significantly reduced by the addition of RCA-p at the high bacterial concentration (Figures-2 and 3). Nevertheless, at lower bacterial concentrations, the addition of RCA-p did not affect bacterial growth. This result could be attributed to the fact that the effect of RCA-p on bacterial growth could not be detected by the conventional methods when the concentration of RCA-p is relatively high.

#### Bacterial growth on MacConkey agar

On MacConkey agar, the growth rate of the bacterial isolates was consistent with that on LB agar. For both bacterial isolates, RCA-p addition resulted in significantly reduced growth at only the higher bacterial concentrations (Figures-4 and 5). This result was consistent with the fact that the relatively high concentration of RCA-p compared to the bacterial concentration in the medium did not alter the bacterial growth. This finding might be attributed to the formation of heteroduplexes of the DNA in the medium rather than interfering with bacteria [26] or the fact that the relatively low concentration of bacteria in the medium could evade the inhibitory effect of RCA-p.

As we did not prepare a standard McFarland bacterial suspension, the precision of the bacterial inhibition result was not highly accurate. In this, we simulated the disk diffusion assay [15,16].

## Conclusion

We confirmed that DNA aptamers, in the form of RCA, were capable of reducing bacterial growth. However, lower concentrations of the bacteria in the samples had no effect, which could be due to the low concentration of RCA as we had no method to calibrate the number of bacteria in the sample with the concentration of RCA. The significance of our study is that RCA-p are very good candidates that could replace traditional antibacterial agents. However, this technique may require a few adjustments to sustain the ability and practicality necessary for commercial requirements.

## Authors’ Contributions

ASA and SSH: Conceived and designed the experiment and conducted and analyzed the data. ASA, SSH, and NN: Contributed to the manuscript drafting and revisions. All authors have read and approved the final manuscript.
